# Integration of One Health education into the medical curriculum

**DOI:** 10.3389/fmed.2026.1820523

**Published:** 2026-04-08

**Authors:** Jourdan K. P. McMillan, Simone A. Evett, Martina L. Kamaka, Sandra P. Chang

**Affiliations:** 1 Department of Tropical Medicine, Medical Microbiology, and Pharmacology, John A. Burns School of Medicine, University of Hawai’i at Mānoa, Honolulu, HI, United States; 2 John A. Burns School of Medicine, University of Hawai’i at Mānoa, Honolulu, HI, United States; 3 Department of Native Hawaiian Health, John A. Burns School of Medicine, University of Hawai’i at Mānoa, Honolulu, HI, United States

**Keywords:** interdisciplinary, medical education, One Health, problem-based learning, transdisciplinary

## Abstract

Health professional training must acknowledge the multifaceted and complex health challenges the global community faces. One Health is an integrated, unifying approach that aims to sustainably balance and optimize the health of people, animals, and ecosystems. One Health competencies can guide health professional training to ensure graduates are prepared for cross-sector collaboration. Several pedagogical approaches to incorporate One Health into the curriculum align with LCME accreditation standards. This manuscript describes One Health educational activities implemented in the medical curriculum at the John A. Burns School of Medicine. By modifying current medical curriculum activities, like problem-based learning and lectures, and introducing new curricular activities, such as interdisciplinary symposia and electives, JABSOM has made significant progress in integrating One Health into the MD curriculum. Student feedback is positive, and assessments suggest students are comprehending and retaining One Health-related content.

## 1 Introduction

The multifaceted and intricate health challenges confronting the global community necessitate healthcare competencies that transcend the conventional medical education framework (1). Health professional training must acknowledge the interconnections leading to various health outcomes and provide foundational competencies for physicians to navigate patient interactions as health providers and trusted sources of information (2–4). The One Health High Level Expert Panel defines One Health as an approach that acknowledges these interconnections: “One Health is an integrated, unifying approach that aims to sustainably balance and optimize the health of people, animals, and ecosystems. It recognizes the health of humans, domestic and wild animals, plants, and the wider environment (including ecosystems) are closely linked and inter-dependent. The approach mobilizes multiple sectors, disciplines and communities at varying levels of society to work together to foster well-being and tackle threats to health and ecosystems, while addressing the collective need for clean water, energy and air, safe and nutritious food, taking action on climate change, and contributing to sustainable development” (5).

The One Health emphasis on cross-sector collaboration requires professionals to have skill sets and knowledge competencies that can facilitate these efforts. To date, there are varying levels of One Health training in health professional schools for veterinarians, public health workers, physicians, and nurses. In veterinary schools, One Health is a central component of the curriculum with numerous One Health courses, institutes and programs. The accrediting body for Public Health schools, the Council on Education for Public Health, includes One Health in its accreditation criteria (6). Accordingly, Master of Public Health and Doctor of Public Health students are expected to be able to “explain an ecological perspective on the connections among human health, animal health, and ecosystem health.” One Health training and education can also be seen in academic degree programs across the United States. The majority of these are Master’s degree programs (60%) that place an emphasis on collaborative work between academia and public health agencies (7). Similar emphasis of One Health in health professional schools is seen internationally (8, 9). The accrediting body for medical education, the Liaison Committee on Medical Education (LCME), does not have explicit standards for incorporating One Health into medical education (10). However, several pedagogical strategies to introduce One Health-related content (11–15) aligns with required LCME standards and accompanying elements (Table 1). Major One Health-related topics that are being incorporated into health professional school curricula include impacts of climate change on human health, antimicrobial resistance, zoonotic diseases, and food and water security. One of the known barriers to incorporating One Health education into medical curricula is the fully packed schedule and limitations for introducing new content (14). Aligning One Health-related topics with required content in the medical curriculum and emphasizing the clinical relevance of One Health can overcome this barrier.

**Table 1:** Strategies to incorporate One Health topics into medical education and their alignment with LCME accreditation standards.

Strategies	LCME accreditation standard
Introduce One Health principles in zoonotic disease coursework	LCME Standard 7.1 “Biomedical, Behavioral, Social Sciences” and 7.4 “Critical Judgment/Problem-Solving Skills”
Incorporate animal contact and environmental exposure histories into the instruction of clinical interviewing	LCME Standard 7.4 “Critical Judgment/Problem-Solving Skills”
Modify clinical cases to emphasize the added value of One Health	LCME Standard 7.4 “Critical Judgment/Problem-Solving Skills”
Facilitate interprofessional activities between medical students and other health professionals in training	LCME Standard 7.7 “Systems-based Practice”
Provide clinical electives at zoos or other relevant facilities	LCME Standard 6.5 “Elective Opportunities”

The John A. Burns School of Medicine (JABSOM) is working toward incorporating elements of One Health into its MD curriculum. Three main learning objectives have been identified for all One Health-related content incorporated into the JABSOM medical curriculum: (1) Define “One Health,” (2) Describe the role of physicians and importance of interdisciplinary collaborations for various One Health issues, and (3) Think holistically about patient health care. The curricular additions include required and elective activities as well as the establishment of One Health-related student interest groups. This report describes additions to the JABSOM medical curriculum to integrate One Health-related topics into medical education.

## 2 Learning environment

The John A. Burns School of Medicine is a 4-year allopathic medical school at the University of Hawai’i at Mānoa. The average class size is 75–80 students. The four-year MD curriculum includes an initial two-year pre-clerkship consisting of problem-based learning, didactics and learning communities which provide curricular content in small group formats as well as community engagement. The pre-clerkship years are divided into 8 to 15-week blocks (MD1-7). The third and fourth year consist of clinical rotations at local hospitals and clinics and in other community settings. Described below are the three main pedagogical approaches to introduce One Health education currently employed at JABSOM. Table 2 lists One Health-related topics, both required and elective, that are covered in the medical education curriculum at JABSOM and the format for delivery of these topics.

**Table 2:** One Health-related topics in JABSOM medical education curriculum.

Year	MD unit	Topics	Delivery format
Year 1	MD1	Climate change and health Heat-related illness Climate change and sea level rise, freshwater inundation and climate migration Zoonotic diseases Vector borne disease	3.5-h symposium 1 h lecture with mini PBL case PBL case PBL and lectures PBL and lectures
	MD2	Antimicrobial resistance Heat-related illness and Cardiovascular disease Respiratory illness and air pollution, increased pollen	PBL case PBL case PBL case (in development)
	MD4	Phthalates and health Zoonotic diseases	PBL case PBL and lectures
	MD5	Key concepts in One Health for medical professionals	4-week introduction to One Health elective course^a^
Year 2	MD6	Climate change and health Vector borne diseases	3.5 h symposium (same as MD1) Lectures
	MD7	Toxoplasmosis	PBL case
Year 4		Clinical practicum elective at the zoo	4-week (~1 h/day) One Health clinical elective course^a^

a Optional elective courses for medical students enrolled in the Certificate of Distinction in One Health.

Problem-based learning (PBL) is a key component of the pre-clerkship medical education curriculum at JABSOM. PBL uses student-led small-group discussions of clinical cases that meet for three hours twice per week and are overseen by faculty tutors. Each clinical case begins with a patient with a chief complaint. As cases are discussed, additional information is revealed about each patient. During the sessions, students identify important topics and concepts and develop a list of learning issues. After completing the clinical case, students research these learning issues and teach each other by presenting to the group. Testable learning objectives are provided at the end of each PBL case to ensure students are comprehending key concepts and knowledge competencies. Strengths of problem-based learning include enhancing critical thinking, applying knowledge to real-life cases thereby promoting knowledge comprehension and the ability to modify cases to incorporate or highlight new content. Weaknesses of problem-based learning include a reliance on student participation and engagement, variability with faculty tutor skill, and the time-consuming process required. Incorporating One Health-related topics by modifying clinical PBL cases aligns with the LCME standard 7.4 “Critical Judgment/Problem-Solving Skills”. Briefly, the incorporation of One Health-related content into existing PBL cases was pitched to the Pre-Clerkship Committee that oversees the first two years of medical training. Following the initial pitch, individual meetings with course directors were held to collaboratively identify where One Health-related content would fit best. To date, PBL cases have been modified to include climate change-related topics such as sea level rise, fresh water inundation, heat-related illness; antimicrobial resistance; zoonotic disease; vector borne disease; and phthalates and environmental pollutants. We are continuing to identify other PBL cases that can be modified to highlight One Health issues. A priority topic is microplastics and pregnancy. Additionally, learning issues for select PBL cases include taking animal and environmental exposure histories, a strategy to integrate One Health into the medical curriculum that aligns with the LCME standard 7.4 “Critical Judgment/Problem-Solving Skills.”

Lectures or didactic learning are utilized for teaching foundational science concepts and clinical knowledge by experts in the field. Traditional and interactive lectures are distributed throughout the pre-clerkship medical education curriculum. One Health-related topics introduced and reviewed in lectures include climate change, heat-related illness, zoonotic diseases, and vector borne diseases. Zoonotic disease lectures can highlight the interconnectedness of human and animal health in a shared environment. Having animal health and environmental health experts provide guest lectures aligns with LCME Standard 7.1 “Biomedical, Behavioral, Social Sciences” and LCME Standard 7.4 “Critical Judgment/Problem-Solving Skills”. Strengths of didactic learning include teaching by content experts and structured, consistent content delivery. Weaknesses of didactic learning include a traditionally passive format, optional student attendance, and low student attention for longer sessions. Additionally, it is challenging to find the space to incorporate new lectures into the already full curriculum. At JABSOM, the first unit of medical school, MD1, covers “Health and Illness” which serves as an introductory unit. One 1-hour lecture introducing heat-related illness was incorporated. While it is challenging to find space for new lectures, the course directors for MD1 are more open to incorporating content that is not organ-specific (for example, Heat-related illness).

Two interdisciplinary workshops and symposia have been developed and introduced into the JABSOM MD curriculum. A required three-and-a-half-hour Climate Change and Health symposium for the first- and second-year medical students and an optional One Health interprofessional conference hosted by student interest groups are held annually. These activities emphasize interprofessional collaboration with professionals across the One Health sectors and align with the LCME Standard 7.7 “Systems-based Practice.” Strengths of interdisciplinary workshops and symposia include promoting collaboration and learning across disciplines. Weaknesses include limited time to cover a wide range of topics, speaker availability constraints, and the large student and faculty time commitment for preparing and executing these types of activities.

One Health training is offered as an elective during the Year 4 clerkship curriculum in partnership with the Honolulu Zoo under the supervision and mentorship of the head veterinarian of the zoo’s Animal Health Center. This practicum course provides students with firsthand experience caring for a variety of animal species presenting with a range of acute and chronic diseases. Students study the physiological similarities and differences between humans and other animals. They also explore the relationships between allopathic and veterinary medicine. As a culminating experience, students complete a clinical or research project over 4 weeks that examines One Health from both the veterinary and human medical perspectives. Strong positive feedback has been obtained from both faculty and students who have participated in this elective.

In addition to coursework, several One Health extracurricular activities are offered sponsored by student interest groups. This includes interest groups and interprofessional education with the School of Nursing. Many of these extracurricular activities address several of the pedagogical approaches (Table 1) with the notable addition of interprofessional activities. The One Health interest group is a medical student-led group that hosts guest speakers and provides opportunities to educate other students about One Health. The interest group also organizes community outreach activities that deepen students’ knowledge and communication of One Health principles. The MS4SF (Medical Students for a Sustainable Future) is another medical student-led group that is responsible for organizing the annual climate change and health symposium. The One Health interest group has collaborated in joint activities with the MS4SF (Medical Students for a Sustainable Future) and the Indigenous Health Interest group (Ka Lama Kukui, KLK). Table 3 includes a representative list of student interest group activities.

**Table 3:** Representative summary of One Health-related student interest group activities.

Interest group	Activity	Description
One Health	One Health symposium	Presentations from veterinarians, as well as case study breakout sessions on One Health topics
	One Health conference	Interprofessional conference with keynote speaker and breakout group discussions with professionals from human medicine, animal science, veterinary medicine, plant sciences, biomedical science, pharmacy, and community. More detail described in text.
	Community outreach events	Various throughout the academic year Examples include beach cleanups with NOAA Hawaii Marine Mammal Response and guided tours with the Honolulu Zoo
	General meetings	Held several times a year to introduce students to the concept of One Health, organize interest group activities, and provide information about the COD program
MS4SF	Climate change and health symposium	Climate and health-focused panel presentations from experts in various fields 2023: Physicians presented on the topics of (1) climate change and the impact on heat illness and skin cancer and (2) climate change and Mauli Ola (Native Hawaiian concept of wellbeing) 2024: Biomedical scientist, mental health professional, and fourth year medical students presented on the health impacts of the 2023 Lahaina wildfires. Small group project: Evaluating various local community climate change impacts. 2025: PhD climate scientist presented on climate change and health and a campus planting, beautification project.
	Planetary health report card	Annual student-completed metric-based tool evaluating JABSOM’s planetary health content and identifying opportunities for improvement in the following areas: planetary health curriculum, interdisciplinary research, institutional support, community outreach and advocacy, campus sustainability
	Community outreach events	Various throughout the academic year Examples include touring a local, sustainably-built, health clinic (collaboration with KLK), Native Hawaiian healing plant garden cleanups, and climate change and health-related film screenings for JABSOM community

## 3 First annual interprofessional One Health conference

The One Health Interest Group sponsors an interprofessional conference modeled after the Zoobiquity conferences which bring together leaders in human and animal health, evolution and conservation biology, and ecology and public health. Zoobiquity conferences aim to address high impact human and animal health concerns through focused collaboration and discussion (16). Similarly, the first JABSOM “One Health in Hawaii” Conference was focused on interprofessional collaboration and featured a keynote speaker, Dr. Katey Pelican, who spoke on “Operationalizing One Health: Fostering collaborative solutions to improve the health and well-being of people, the planet, and all living things.” This presentation was followed by small group discussions of three One Health-related scenarios. The discussion groups included students and professionals in human medicine, veterinary medicine, animal science, basic science research, public health, pharmacy, community research, and representatives of non-profit organizations. Each participant considered the scenario from their own perspective and contributed to an exchange of ideas to identify approaches and challenges in addressing One Health issues. A post-conference survey indicated that the scenario discussions enabled participants to gain new insights into the One Health approach to address human, animal, and environmental health issues. Furthermore, survey respondents indicated that interprofessional connections were established and may lead to future educational or research collaborations.

## 4 Evaluation

For One Health-related content delivered to medical students, surveys are the main assessment for evaluating student feedback, content comprehension and retention. Surveys were administered to first year medical students before and after a required one-hour lecture and case discussion about heat-related illness offered in 2024 and 2025. The survey results are summarized in Table 4. There is variability between years where some have heard of One Health and others have not. Interestingly, for those that responded “yes” to the question “Have you heard of One Health before?”, the majority referenced hearing about One Health during the first unit of medical school at the medical student activity fair, a One Health interest group meeting or Dean’s Certificate of Distinction informational sessions. To determine whether students are retaining heat-related illness knowledge during these multidisciplinary presentations, we compared pre- and post-session survey responses to questions about heat-related illness. The majority of post-session survey respondents answered the question correctly as compared to pre-session survey respondents. After the presentation, 97–98% of the respondents agreed or strongly agreed that they had a better understanding of climate change and the impacts on health and heat-related illness. Long-term follow up is ongoing since these activities have only recently been introduced. We plan to survey the fourth-year medical students about One Health-related content. An opportunity to hold graduation focus groups may also be possible.

**Table 4:** Survey results summary for One Health-related lecture.

	2024 (*n* = 37)		2025 (*n* = 61)	
Survey Question	Survey Responses			
Have you heard of One Health before?	Yes/maybe	No	Yes/maybe	No
	Percent Responses in Each Category (number of responses)			
	59.5% (22)	40.5% (15)	32.8% (20)	57.4% (35)
Survey Question	Time of Survey Administration (number of respondents)			
Which of the following is not a type of heat related illness?	Pre^*^ (*n* = 37)	Post (*n* = 32)	Pre (*n* = 61)	Post (*n* = 59)
	Correct Responses: Percentage correct (number correct)			
	13.5% (5)	56.3% (18)	26.2% (16)	71.2% (42)
^*^Percentage with the correct answer; Pre = before the lecture, Post = after the lecture.				

After this presentation, I have a better understanding of climate change and the impacts on health and heat-related illness (1 = strongly disagree, 5 = strongly agree).					
	1	2	3	4	5
2024 (*n* = 32)	0	0	3.1% (1)	21.9% (7)	75% (24)
2025 (*n* = 59)	0	0	1.7% (1)	27.1% (16)	71.2% (42)

## 5 Discussion

JABSOM has made significant progress in integrating One Health-related content and activities into the MD curriculum through the adoption of several pedagogical approaches. These included symposia, lectures, integration of One Health content into PBL cases, a didactic elective course, and a clinical practicum course. Concepts presented through these pedagogical approaches align with current LCME accreditation standards, providing continued and lasting assimilation of One Health education throughout all 4 years of the MD curriculum.

One of the major barriers to implementing One Health-related content in medical education is the lack of available space in the curriculum (14, 17). The approach taken by this program has been to provide opportunities for One Health education for all students through the integration of key concepts into the existing problem-based learning curriculum and symposia. Medical students with a particular interest in One Health also have an opportunity to participate in a One Health Interest Group which engages them in extracurricular activities that provide One Health education and outreach to their peers and the community. Individuals having a higher level of commitment can enroll in the Certificate of Distinction in One Health, a four-year program which provides them with focused One Health coursework, a One Health practicum course experience, and an opportunity to complete a capstone clinical or research One Health project (18).

In a 2020 survey of 26 LCME accredited medical education programs, 54% included some form of One Health related content in their curriculum covering subject matter such as infectious diseases, zoonotic diseases, emergency preparedness, foodborne illness, patient safety and living environment assessment (19). Around half of the surveyed institutions that offered One Health subjects matter responded that it was “very important” or “important” to include One Health into medical school curricula. Major barriers to the introduction of One Health training into medical education were that the curriculum is already too full and that there was a lack of expertise to teach One Health. The lack of education and training of One Health practitioners and educators was also cited in a previous systematic review (20). The presence of faculty with global infectious disease expertise in the Department of Tropical Medicine, Medical Microbiology and Pharmacology at the John A. Burns School was a key enabler for the development of the One Health curriculum. This expertise is complemented with that of faculty from the Department of Native Hawaiian Health who possess background knowledge in climate change, sustainability, and cultural competency. Other contributors to the curriculum include faculty from the Office of Public Health Studies in the Thompson School of Social Work and Public Health and from the Department of Human Nutrition, Food and Animal Sciences in the College of Tropical Agriculture and Human Resilience. This multidisciplinary expertise drawn from different departments within and outside of the medical school greatly enhanced the development and implementation of the One Health medical education curriculum.

A review of the integration of One Health principles by the Global One Health Partnership established to advance interdisciplinary research and education have analyzed the strengths and weaknesses of pedagogical approaches in One Health education (9). Many of the approaches described in this study have been incorporated into the JABSOM One Health curriculum, including problem-based learning, case-based learning, and interdisciplinary workshops. An additional approach that may be developed in the future at JABSOM is simulation-based education which proves hands-on, experiential learning and prepares participants for professional practice. There is an opportunity to develop this approach through the JABSOM SimTiki Simulation Center (21) which already provides workshops on interprofessional education using interactive learning and simulation. In addition to providing a realistic environment for practicing clinical skills, the simulation platform may provide an opportunity for the evaluation of student achievement of One Health learning objectives.

In conclusion, the integration of One Health education at JABSOM is evident. An interdisciplinary team has made the development and implementation of One Health-related activities and coursework successful. The JABSOM One Health curriculum has addressed major barriers to implementing One Health education. Future evaluations of One Health medical education at JABSOM will include follow up of One Health knowledge and competency retention and long-term follow up of the impact of One Health education on interprofessional engagement and patient care after graduation into medical practice.

## Data Availability

The original contributions presented in the study are included in the article/supplementary material, further inquiries can be directed to the corresponding author.
